# The Bile Acid Sensor FXR Is Required for Immune-Regulatory Activities of TLR-9 in Intestinal Inflammation

**DOI:** 10.1371/journal.pone.0054472

**Published:** 2013-01-25

**Authors:** Barbara Renga, Andrea Mencarelli, Sabrina Cipriani, Claudio D'Amore, Adriana Carino, Angela Bruno, Daniela Francisci, Angela Zampella, Eleonora Distrutti, Stefano Fiorucci

**Affiliations:** 1 Dipartimento di Medicina Clinica e Sperimentale, Università degli Studi di Perugia, Perugia, Italy; 2 Dipartimento di Medicina Clinica e Scienze Biochimiche, Università degli Studi di Perugia, Perugia, Italy; 3 Dipartimento di Chimica delle Sostanze Naturali, Università di Napoli “Federico II”, Napoli, Italy; 4 Azienda Ospedaliera di Perugia, Ospedale Santa Maria della Misericordia, Perugia, Italy; University of Chicago, United States of America

## Abstract

**Background:**

Toll like receptors (TLRs) sense the intestinal microbiota and regulate the innate immune response. A dysregulation of TLRs function participates into intestinal inflammation. Farnesoid X Receptor (FXR) is a nuclear receptor and bile acid sensor highly expressed in entero-hepatic tissues. FXR regulates lipid metabolism and innate immunity.

**Methodology/Principal Findings:**

In this study we have investigated whether FXR gene expression/function in the intestine is modulated by TLRs. We found that in human monocytes activation of membrane TLRs (i.e. TLR2, 4, 5 and 6) downregulates, while activation of intracellular TLRs (i.e. TLR3, 7, 8 and 9) upregulates the expression of FXR and its target gene SHP, small heterodimer partner. This effect was TLR9-dependent and TNFα independent. Intestinal inflammation induced in mice by TNBS downregulates the intestinal expression of FXR in a TLR9-dependent manner. Protection against TNBS colitis by CpG, a TLR-9 ligand, was lost in FXR^−/−^ mice. In contrast, activation of FXR rescued TLR9^−/−^ and MyD88^−/−^ mice from colitis. A putative IRF7 response element was detected in the FXR promoter and its functional characterization revealed that IRF7 is recruited on the FXR promoter under TLR9 stimulation.

**Conclusions/Significance:**

Intestinal expression of FXR is selectively modulated by TLR9. In addition to its role in regulating type-I interferons and innate antiviral immunity, IRF-7 a TLR9-dependent factor, regulates the expression of FXR, linking microbiota-sensing receptors to host's immune and metabolic signaling.

## Introduction

Innate immunity is central to host defense against invading pathogens, providing recognition of microorganisms and rapid deployment and activation of effector cells [1]. Activation of innate immunity also initiates subsequent adaptive immune responses. The ability to recognize microorganisms depends in part on a family of receptors known as the Toll-like receptors (TLRs) [1], [2]. There are 13 known mammalian TLRs. Ligand engagement of TLR leads to activation of two pathways. TLR1, 2, 4, 5, 6, 7, 8, and 9 signal via the MyD88 adaptor, whereas TLR3 activates an alternative “MyD88-independent” pathway [1], [2]. TLR4 is the only receptor known to activate both MyD88 dependent and independent pathways [1], [2].

TLRs can be divided into two groups on the basis of their subcellular localization: TLR1, 2, 4, 5 and 6 are expressed on the surface of the cells and recognize lipid structures and in the case of TLR5, the protein flagellin. TLR3, 7, 8 and 9 all reside intracellularly and recognise nucleic acids. The localization and trafficking of TLRs within the cell is an important mechanism to allow TLRs to sense proper ligands and modulate downstream signaling [1], [2]. A body of evidence support a mechanistic role of TLR dysfunction in development of inflammatory bowel diseases (IBDs) [3].

Nuclear receptors are transcription factors highly expressed in entero-hepatic tissues integrating nutrient absorption, lipid and glucose metabolism, energy homeostasis, reproduction and development, and xenobiotic metabolism [4], [5]. There is evidence that these transcription factors undergo gene regulation in response to the microbial flora residing in the gastrointestinal tract and that this changes contributes to local development and tuning of gut homeostasis in addition to driving maturation of the host adaptive immune system [6]–[8].

Recent data suggest that nuclear receptors are regulated under intestinal inflammation [9], [10]. This view emerges from the observation that while commensal bacteria elevate the expression of peroxisome proliferator-activated receptor (PPAR)γ in colonic epithelial cells and can regulate intestinal inflammation by inhibiting NF-kB activity in a PPARγ-dependent manner [11], inflammation induced in rodents or by IBDs associates with a robust downregulation of the expression of a number of nuclear receptors including PPARγ, liver-x-receptor (LXRs), pregnane-x-receptor (PXR), farnesoid-x-receptor (FXR) and retinoid-x-receptor (RXR) among others [9]. Because these receptors exert counter-regulatory activities on macrophages and epithelial cells by inhibiting downstream targets of the TLR pathways [12], [13], aberrations in their expression might have impact in the pathogenesis of human diseases. Further on, because nuclear receptors exert their regulatory effects beyond the intestinal wall, their dysregulation might have systemic effects.

FXR is a bile acid sensor whose expression is highly restricted to entero-hepatic tissues [14]. FXR is required to maintain intestinal integrity and its deficiency results in altered intestinal permeability and tendency toward development of dysregulated immune response [15], [16]. Despite a dysregulated expression of FXR has been linked to IBDs [17], the mechanisms that govern FXR expression in the intestine are poorly defined.

In the present study we have investigated the mechanism of regulation of FXR by TLRs. By using mice deficient for several TLRs we have obtained compelling evidence that FXR is a downstream effector of immune response triggered by TLR9. In addition, we have provided evidence that modulation of FXR by TLR-9 is mediated by the recruitment of interferon regulatory factor (IRF)-7, linking microbiota-sensing receptors to immune and metabolic signaling in the intestine.

## Results

### FXR is differentially regulated by TLRs in monocytes

**Figure 1:**
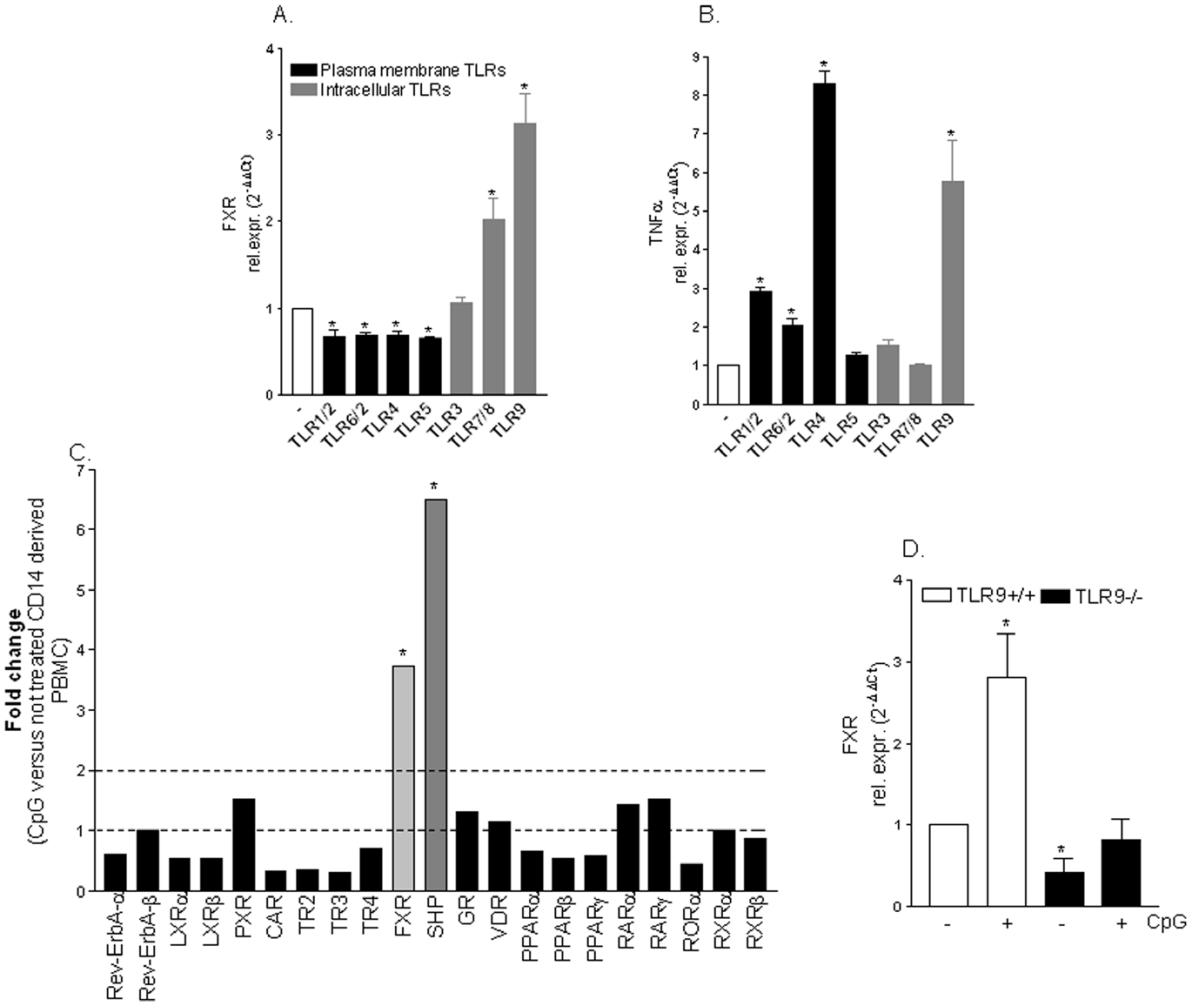
FXR gene expression is regulated by TLR agonists. (A–B) Quantitative RT-PCR of FXR and TNFα genes was carried out on RNA purified from CD14 positive cells derived PBMC stimulated *ex vivo* with TLRs agonists as described in the materials and methods. Data are mean ± SE of 3 experiments carried out in triplicate. *P<0.05 versus not treated cells. (C) Specificity of CpG effect. PCR array analysis showing the relative mRNA expression of various nuclear receptors on CD14^+^ derived PBMC stimulated *ex vivo* with TLR9 agonist CpG. (D) Quantitative RT-PCR of FXR was carried out on RNA purified from spleen-derived monocytes isolated from TLR9^+/+^ and TLR9^−/−^ mice stimulated *ex vivo* with CpG. Data are mean of ± SE of 4 mice. *P<0.05 versus TLR9^+/+^ not treated cells.

We have first investigated whether expression of FXR gene is regulated by TLRs agonists. For this purpose, CD14 derived PBMC were stimulated *ex vivo* with ligands for TLR1–9. As shown in Figure 1 A, the activation of extracellular TLRs (i.e TLRs 1/2, 2/6, 4 and 5) caused a significant down-regulation of FXR gene expression (Figure 1 A; n = 3; p<0.05). In contrast, while exposure of monocytes to TLR3 agonist had no effect on FXR expression, exposure of PMBC-derived monocytes to TLR7/8 and TLR9 ligands resulted in ∼3 fold induction of FXR mRNA (Figure 1 A; n = 3; p<0.05). These effects were independent on the ability of TLRs ligand to modulate TNFα mRNA since both TLR4 and TLR9 ligands increased TNFα mRNA expression (Figure 1 B; n = 3; p<0.05). Since exposure of monocytes to TNFα, *per se*, down-regulates FXR gene expression [18], these data demonstrated that the effect exerted by TLR9 on FXR gene expression is direct.

### TLR9 selectively targets FXR and its target gene Small Heterodimer Partner (SHP)

To further investigate on the specificity of the effects exerted by TLR9 on FXR gene expression we applied a PCR array to analyze the relative mRNA expression of several nuclear receptors on CD14 derived PBMC treated with the TLR9 agonist CpG. As illustrated in Figure 1C, exposure of human monocytes to the TLR9 agonist CpG resulted in a selective induction of FXR mRNA and its target gene SHP, while expression of other nuclear receptors was unchanged (Figure 1 C; n = 3, p<0.05). The specificity of this interaction was further confirmed by *ex vivo* CpG stimulation of spleen-derived monocytes isolated from TLR9^+/+^ and TLR9^−/−^ mice. Results of Real-Time PCR confirmed that CpG effectively induced FXR mRNA expression in TLR9^+/+^ spleen derived monocytes but not in TLR9^−/−^ cells (Figure 1 D; n = 4;p<0.05).

### Severity of colitis induced by TNBS is modulated by expression of TLRs and FXR

**Figure 2:**
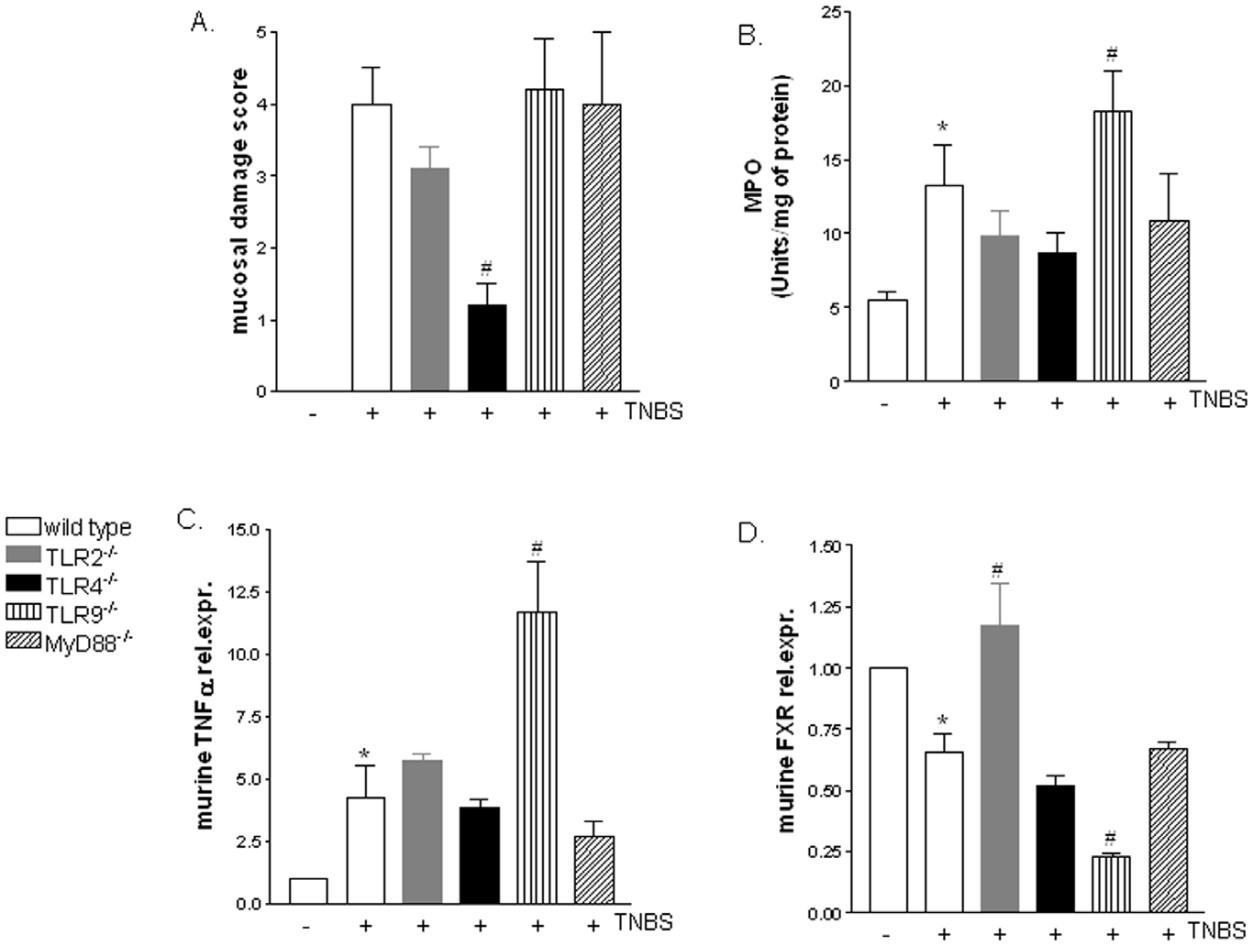
FXR is down-regulated in TLR9^−/−^ mice administered TNBS and upregulated in TLR2^−/−^ mice administered TNBS. TNBS colitis was induced in C57BL/6, MyD88^−/−^ TLR2^−/−^, TLR4^−/−^ and TLR9^−/−^ mice as described in materials and methods section. (A) Mucosal damage score, (B) myeloperoxidase activity (MPO), (C–D) quantitative RT-PCR of TNFα and FXR genes. In all panels data rare the mean ± SE of 6 animals. *P<0.05 versus naive mice. # P<0.05 versus TNBS wild type.

Since FXR is a robust mediator of innate host defense in the intestine [3] and colon expression of FXR mRNA is reduced in IBDs [19] we have next investigated the colonic expression of FXR in a murine model colitis induced by TNBS administration to wild type (C57BL/6) and TLR2^−/−^, TLR4^−/−^, TLR9^−/−^ and MyD88^−/−^ mice. As shown in Figure 2, analysis of mucosal damage score demonstrated that, with the exception of TLR4^−/−^ mice which showed a less severe disease, the severity of the colitis was essentially similar in wild type, TLR2^−/−^, TLR9^−/−^ and MyD88^−/−^ mice (Figure 2 A; n = 6;p<0.05). However, a tendency toward a development of more severe disease was observed in mice lacking TLR9, as demonstrated by higher colonic myeloperoxidase (MPO) activity (Figure 2 B; n = 6, p<0.05) and TNFα levels (Figure 2 C; n = 6; p<0.05). Of interest, compared to C57BL/6 mice administered TNBS, the relative expression of FXR mRNA was strictly down-regulated in TLR9^−/−^ mice (Figure 2 D; n = 6, p<0.05), confirming in vitro experiments indicating that TLR9 exerts a positive effect in regulating the FXR gene expression.

### FXR activation protects against colitis development in TLR9 and MyD88 null mice

**Figure 3:**
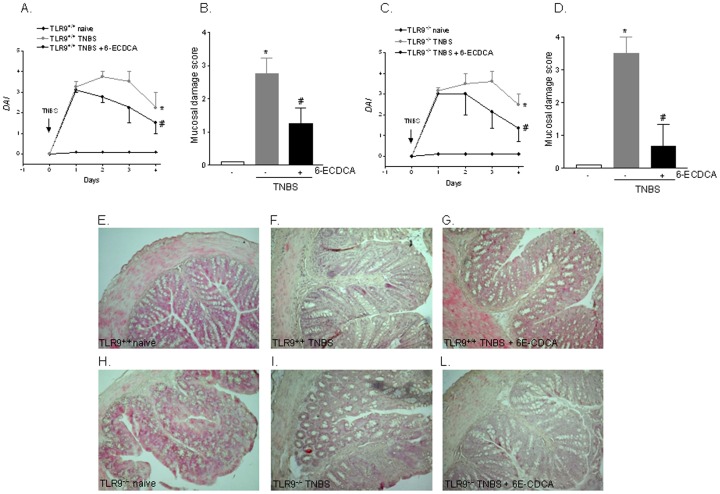
FXR agonism protects against colitis development in TLR9^−/−^ mice. TNBS colitis was induced in TLR9^+/+^ and TLR9^−/−^ mice. Mice were administered 6-ECDCA, a FXR agonist, as described in materials and methods. (A–C) Analysis of Disease activity index (DAI) in TLR9^+/+^ (A) and TLR9^−/−^ mice (C). (B–D) Analysis of mucosal damage score in TLR9^+/+^ (B) and TLR9^−/−^ mice (D). (E–L) H&E staining of representative paraffin-embedded sections from distal colons after administration of vehicle (control mice), TNBS or TNBS plus 6-ECDCA in TLR9^+/+^ (panels E–G) and TLR9^−/−^ (H–L) mice. Data are mean ± SE of 6 animals.*P<0.05 versus wild type naive mice. #P<0.05 versus wild type mice administered TNBS.

**Figure 4:**
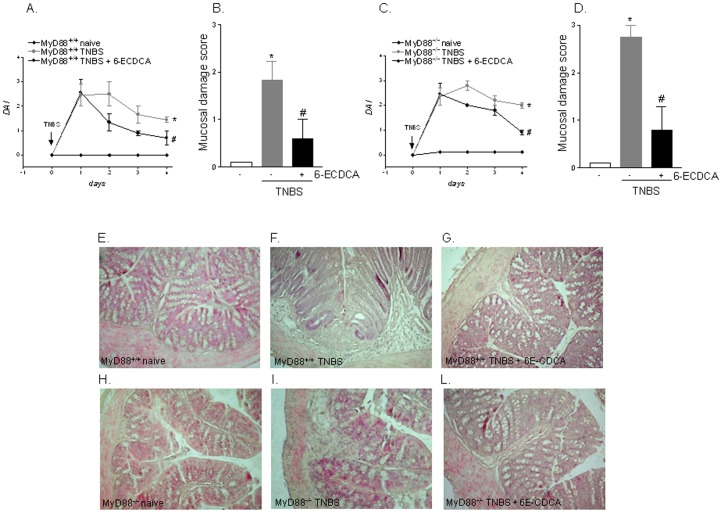
FXR agonism protects against colitis development in MyD88^−/−^ mice. TNBS colitis was induced in MyD88^+/+^ and MyD88^−/−^ mice. Mice were administered 6-ECDCA, a FXR agonist, as described in materials and methods section. (A–C) Analysis of Disease activity index (DAI) in MyD88^+/+^ (A) and MyD88^−/−^ mice (C). (B–D) Analysis of mucosal damage score in MyD88^+/+^ (B) and MyD88^−/−^ mice (D). (E–L) H&E staining of representative paraffin-embedded sections from distal colons after administration of vehicle (control mice), TNBS or TNBS plus 6-ECDCA in MyD88^+/+^ (panels E–G) and MyD88^−/−^ (H–L) mice. Data are mean ± SE of 6 animals.*P<0.05 versus wild type naive mice. #P<0.05 versus wild type mice administered TNBS.

Since activation of TLR9 transduces its signal by recruiting the adaptor molecule MyD88 (Figure S1) we have then investigated the role exerted by FXR on development of TNBS colitis in TLR9^−/−^ and MyD88^−/−^ mice. As illustrated in Figure 3 and 4, the analysis of disease activity index (DAI) and mucosal damage score demonstrates that activation of FXR by 6-ECDCA effectively rescued against the development of local and systemic signs and TNBS colitis. The extent of this protection was comparable among wild type and TLR9^−/−^ and MyD88^−/−^ mice indicating that TLR9 and its target adaptor molecule MyD88 were not involved in the protective effects of FXR (Figure 3 and 4; n = 6; p<0.05). All together these results intracellular signaling activated by FXR lies downstream to TLR9 and MyD88.

### The TLR9 agonist CpG fails to protect against the development of TNBS colitis in FXR^−/−^ mice

**Figure 5:**
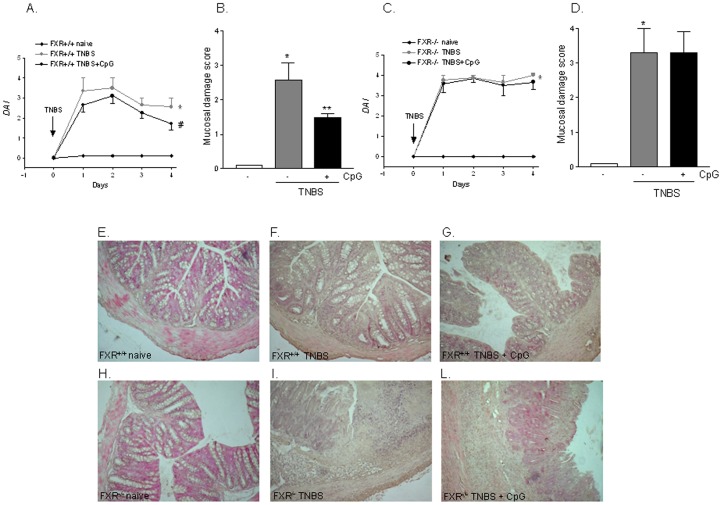
An intact FXR signalling is required to preserve TLR9 action. TNBS colitis was induced in FXR^+/+^ and FXR^−/−^ mice. Mice were administered CpG as described in materials and methods. (A–C) Analysis of Disease activity index (DAI) in FXR^+/+^ (A) and FXR^−/−^ mice (C). (B–D) Analysis of mucosal damage score in FXR^+/+^ (B) and FXR^−/−^ mice (D). (E–L) H&E staining of representative paraffin-embedded sections from distal colons after administration of vehicle (control mice), TNBS or TNBS plus CpG in FXR^+/+^ (panels E–G) and FXR^−/−^ (H–L) mice. Data are mean ± SE of 6animals.*P<0.05 versus naive mice. #P<0.05 versus mice administered TNBS.

Because previous studies have shown that TLR9 activation exerts protective effects against the development of colitis [20], [21] we have investigated whether TLR9 activation by *in vivo* administration of CpG rescues FXR^−/−^ mice from colitis induced by TNBS administration. Of interest, while CpG effectively protected FXR^+/+^ mice against development of colitis, it had no effect on severity of TNBS colitis in FXR^−/−^ mice (Figure 5; n = 6; p<0.05), suggesting that FXR is a non-dispensable component of the protective mechanism activated by TLR9 in this model.

### Analysis of human and mouse FXR promoters reveals the existence of a conserved IRF-7 responsive element (IRF-7RE)

**Figure 6:**
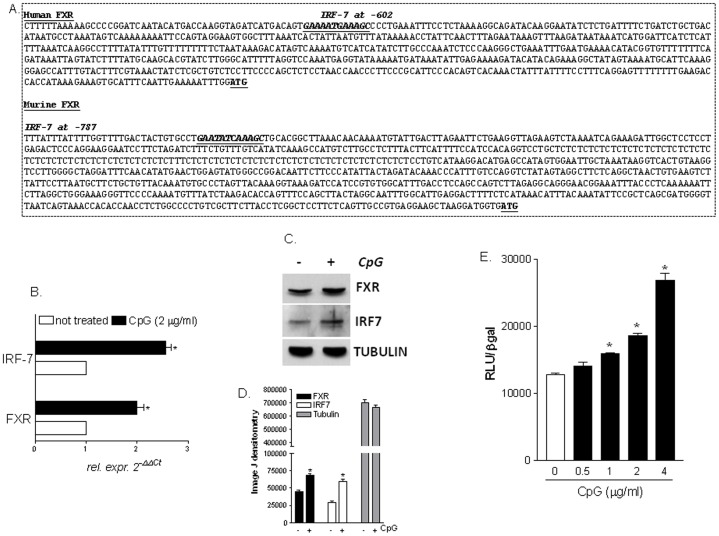
A conserved IRF7 responsive element is expressed in the promoter of FXR. (A) Analysis of the FXR promoter was performed with the on-line software TFsearch. The human FXR 5'flanking region contains an IRF7-RE at −602 base pairs with respect to the transcriptional start site ATG. The murine FXR 5'flanking region contains an IRF7-RE at −787 base pairs with respect to the transcriptional start site ATG. (B) Quantitative RT-PCR of FXR and IRF7 genes was carried out on RNA purified from Raw264.7 cells stimulated with CpG. Data are mean ± SE of of 4 experiments. *P<0.05 versus not treated cells. (C) Western Blotting analysis of FXR, IRF7 and tubulin was performed on protein extracts from Raw264.7 cells stimulated with CpG. The image shown is one of three showing the same pattern. (D) Densitometric analysis of Western blot bands carried out using the Image J software. Data are the mean of three experiments. (E) Transactivation of IRF7-RE. Three copies of the murine IRF7-RE were cloned into the luciferase reporter vector pGL4. Raw264.7 cells were transiently transfected with this construct and forty-eight hours post-transfection cells were stimulated with increasing concentrations of CpG. Cellular extracts were subsequently assayed for luciferase activity. Data are the mean ± S.E. of 3 experiments carried out in triplicate. *P<0.05 versus not treated cells.

Signaling to TLR9-MyD88 activation requires the recruitment of the transcription factor IRF7 (Figure S1), which, in turn, binds to responsive elements (RE) in the promoter of target genes enabling the transcription of type-I interferons [22]. We have therefore searched for putative IRF-7 responsive elements (IRF7-REs) in the promoter of FXR. The analysis of 5'flanking region of both human and mouse FXR gene carried out with the on-line software TFsearch revealed the presence of a conserved IRF7-RE located at −602 base pairs in the human FXR gene and at −787 base pairs in the murine FXR gene, with respect to the transcriptional start site ATG (Figure 6A). For practical reasons, i.e. ease of transfection, rapid replication and achievement of high number of cells to perform molecular experiments such as Chromatin Immunoprecipitation (ChIP) and Electrophoretic Mobility Shift Assay (EMSA), we have decided to assess the functionality of this IRF7RE sequence in Raw264.7 cells. As shown in Figure 6B, C, D, exposure of Raw264.7 cells to CpG resulted in a ∼2 folds induction of FXR and IRF7, mRNA and protein (Figure 6 A; n = 3, p<0.05). To explore the functional role of this putative IRF7-RE, three copies of the IRF7-RE were cloned in the pGL4 vector (pGL4 (IRF7RE)_3X_). Using this reporter vector we have then investigated whether the identified IRF7-RE confers responsiveness to CpG stimulation with a luciferase reporter gene assay. For this purpose, a concentration-response curve was generated exposing Raw264.7 transiently transfected with the pGL4(IRF7RE)_3X_ to CpG. As illustrated in Figure 6E, the effect of CpG on the induction of the reporter gene expression was concentration-dependent with an EC_50_ of ∼2 µg/ml (Figure 6E; n = 3; P<0.05).

### IRF-7 physically interacts with the FXR promoter after TLR9 activation

**Figure 7:**
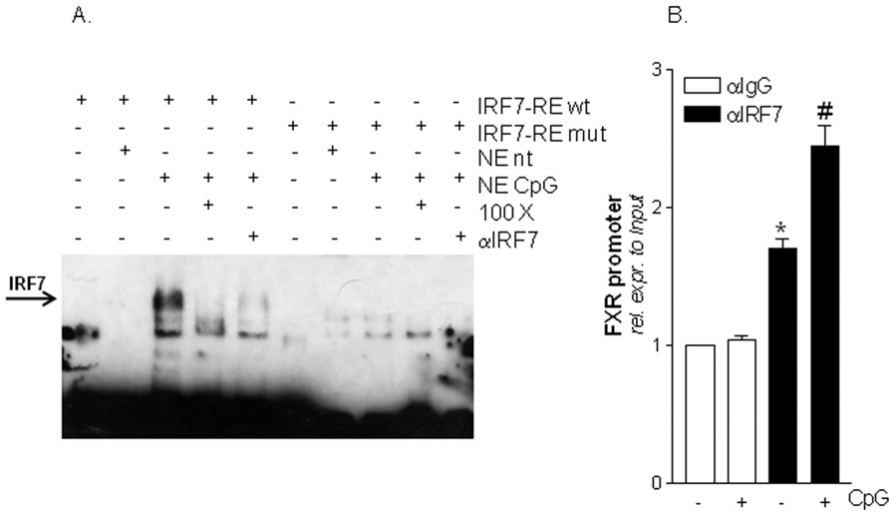
IRF7 binds to the an IRF7-RE located in the FXR promoter. (A) Electrophoretic Mobility shift assay (EMSA). Nuclear extracts from Raw264.7 cells left untreated or stimulated with CpG were incubated in the presence of a wild type or a mutated IRF7 biotin-labeled probe. Competition experiments were performed with a 100 fold excess of unlabeled oligo or with 1 µg IRF7 antibody. (B) Chromatin immunoprecipitation (ChIP). ChIP assay carried out in Raw264.7 cells left untreated or primed with CpG as described in materials and methods section. Values are normalized relative to input DNA concentration and are expressed relative to those of not treated cells immunoprecipitated with an anti IgG antibody, condition set as 1. Analysis was carried out in triplicate and the experiment was repeated twice. *P<0.05 versus not treated cells immunoprecipitated with an anti-IgG antibody. #P<0.05 versus not treated cells immunoprecipitated with an anti-IRF7 antibody.

To verify the hypothesis that IRF7 binds the putative IRF7-RE in the FXR promoter, we have then performed an EMSA experiment using the following biotin-labelled probes: IRF7-RE and IRF7RE_mutated_. These probes were incubated with nuclear extracts from Raw264.7 cells not treated or stimulated with CpG. As shown in Figure 7A, the binding of IRF7-RE probe was undetected when this probe was incubated with nuclear extracts from naive cells while the exposure to CpG strongly induced this interaction (Figure 7 A; lanes 2 and 3). In contrast, CpG failed to induce IRF7 binding when an IRF7-RE mutated probe was used (Figure 7 A; lanes 5 and 6). We confirmed the specificity of these interactions by adding 100 fold excess of unlabeled IRF7 probes or 1 µg IRF7 antibody (Figure 7 A; lanes 4, 5 and 9, 10). All these approaches reduced the binding of the IRF7 biotin labelled probe to nuclear extracts from CpG stimulated cells. Finally we have performed a ChIP experiment to further examine the IRF7-FXR promoter interaction within the context of chromatin. For this purpose we have used Raw264.7 cells exposed to CpG. As show in Figure 7B, quantitative Real-Time PCR performed with primers flanking the FXR promoter region containing the IRF7 sequence confirmed the binding of IRF7 to the FXR gene (Figure 7 B; n = 3; P<0.05). Thus, the functionality of this site was further confirmed in the context of intact chromatin structures.

## Discussion

Members of the nuclear-receptor superfamily have well-documented regulatory effects in orchestrating metabolic and inflammatory programs of gene expression in macrophages and epithelial tissues [23]. The ability of nuclear receptors to repress transcriptional responses to diverse signaling pathways is an essential aspect of their biological activities. Previous studies have highlighted a nuclear receptor-specific mechanisms that mediate signal- and gene-specific repression of transcriptional responses initiated by engagement of TLR3, 4 and 9 [24]. Ogawa et al. have shown that inhibition of TLR-4 and TLR-9 activated programs by glucocorticoid receptor (GR) is regulated in a signal-specific manner through the interaction of GR signaling with IFR3- and IRF7-dependent transcriptional activation and by a disruption of the formation of an IRFs/p65 activator/coactivator complex [25].

In contrast, inhibition of TLR4 and 9 by Proxisome Proliferator Activated Receptor (PPAR)-γ was reported to be p65 independent and mediated by prevention of the clearance of the Nuclear Corepressor (NcoR) complex from the promoter of specific set of genes involved in inflammation [12], [13]. A similar mechanism, i.e. stabilization of NCoR to the promoter of Interleukin(IL)-1β, Tumor Necrosis Factor(TNF)-α and inducible nitric oxide synthase (iNOS), mediates counter-regulatory effects of FXR in LPS stimulated macrophages, indicating a common theme for nuclear receptors in regulating TLRs signaling [19].

In the present study we have extended further on the reciprocal regulation between FXR and TLRs by revealing that FXR takes part into a regulatory loop that is specifically activated by TLR-9. Indeed, analysis of the effects exerted by members of human TLR family on FXR gene expression led to the discovery that activation of membrane associated TLRs (i.e. TLR4) in macrophages downregulates the expression of FXR, while activation of TLR9, a prototype of endosomal TLRs, by CpG upregulates the expression of FXR. Importantly the effects exerted by CpG were restricted to FXR, because activation of TLR-9 induces only the expression of FXR and its target gene SHP, but failed to upregulate the expression of other nuclear receptors including PPARs, LXRs, RXR, RAR and GR, among the others. Together with the fact that the regulatory effects exerted by CpG on FXR gene expression were lost in macrophages isolated from the TLR9^−/−^ mice, these data illustrates that TLR9 triggers a specific program that lead to a direct up-regulation of FXR and its target gene SHP. Because SHP interacts and inhibits the transcription factor AP-1 [26], a positive regulator of inflammatory response, these data support the notion that, in addition to regulating the expression of α/β interferons, TLR9 induces an additional regulatory system based on two nuclear receptors, FXR and SHP, that functions as braking signals for inflammation in the intestine. Accordingly, FXR deficient mice develop a proinflammatory and profibrotic phenotype in the intestine and are prone to develop bacterial overgrowth and exacerbated colitis in response to TNBS [15], [16], [19]. Moreover, the survival of these mice in response to TNBS administration is reduced in comparison to C57/BL6 mice and the treatment with an FXR agonist fails to protect against colitis development [19].

The essential role of TLR9/FXR interaction in shaping intestinal immune responses was further investigated by using specific TLRs and FXR deficient mice challenged with TNBS, a model of colonic inflammation mediated by activation of T cells with a TH1 polarization [27]. Results of these studies demonstrate that, in comparison with C57BL6 wild type mice, colitis severity was attenuated by TLR4 ablation, while the opposite was observed in TLR9^−/−^ mice (Table S1).

In contrast, no changes in colitis severity was detected in mice harboring a disrupted TLR2 and MYD88 gene. Noteworthy, in comparison with C57BL6 wild type mice treated with TNBS, the colonic FXR expression was up-regulated in TLR2^−/−^ but not in TLR4^−/−^ mice, despite the colitis was less severe in these mice. Finally, FXR mRNA expression was up-regulated in TLR9^−/−^ mice after TNBS challenge. This finding is consistent with the fact that in comparison with wild type mice, the severity of colitis is exacerbated in TLR9^−/−^ mice. In turn, these findings demonstrate that the differential modulation exerted by various TLRs agonists on FXR gene, especially that exerted by negative modulators of FXR (TLR1/2, TLR6/2 and TLR4 agonists), might also occur with a TLR/MyD88 independent pathway.

The functional relevance of the TLR9/FXR interaction was further investigated by administering TLR9^−/−^and MyD88^−/−^ mice with an FXR agonist. Results of these investigations demonstrate that FXR activation effectively rescued TLR9^−/−^ and MyD88^−/−^ mice from colitis induced by TNBS, strionlgly indicating that the FXR signaling pathways lies downstream to TLR9 and MyD88 and is conserved in mice lacking the expression of these genes.

Previous studies have shown that CpG rescues wild type mice from murine colitis, highlighting a role for TLR9 generated signals in repressing intestinal inflammation [20] and activation of TLR9 is instrumental to the immune-regulatory activity of probiotics in rodent models of colitis [21]. However, since *in vivo* CpG administration failed to rescue FXR^−/−^ from colitis induced by TNBS, it appears that FXR is a non-dispensable component of the immune-modulatory activity of TLR9.

Another important finding of this study was the demonstration that regulation of FXR by TLR9 is mediated by activation of IRF7. IRF7 is a member of the interferon regulatory family of transcription factors involved in the transcriptional activation of virus-inducible cellular genes, including the type I interferon genes [22]. IRF7 is essential for the induction of IFN-α/β genes via the virus-activated, MyD88-independent and the TLR-activated MyD88-dependent pathway [22]. Viral induction of IFN-α/β genes is severely impaired in IRF^−/−^ fibroblasts and IRF^−/−^ mice are more vulnerable than Myd88^−/−^ mice to viral infection, and this correlates with a decrease in serum IFN levels, indicating the importance of the IRF7-dependent induction of systemic IFN responses for innate antiviral immunity [28]. Furthermore, robust induction of IFN production by activation of the TLR9 subfamily is entirely dependent on IRF7 [22]. IRF7-RE have been detected in the promoter of several CpG responsive genes [29]. In the present study we report the detection of a IRF7-RE in the promoter of FXR. This IRF7-RE was conserved across species and its functionality was examined by a variety of molecular approaches. Results from ChIP, EMSA and transactivation assays have shown that not only IRF7 binds to the FXR promoter in response to TLR9 stimulation with CpG, but that this interaction results in a IRF7 mediated transcription of the FXR gene. Furthermore, by EMSA we have demonstrated that IRF7 binds to a specific IRF7 consensus and that mutation of this consensus results in the abrogation of the binding.

Present findings might have a therapeutic relevance because probiotics that are increasingly used for treating IBDs and other intestinal disorders are positive regulator of FXR expression [30]. Since FXR functions as a negative regulator of inflammatory responses, present data uncover a striking mechanism through which nuclear receptors function as a gatekeeper signaling in regulating intestinal immune response to microbiota.

In conclusion, we have shown that a TLR9/MyD88/IRF7 signaling positively regulates the expression of the bile acid sensor FXR in the intestine and that FXR mediates housekeeping activities of TLR9 thus linking microbiota-sensing receptors to immune and metabolic signaling in the intestine.

## Materials and Methods

### Reagents

The “TLR Ligands Set I” and the TLR9 agonist, synthetic oligodeoxynucleotides (ODN), containing unmethylated deoxycytosine-deoxyguanosine (CpG), CpG ODN 2395, were from Apotech Corporation. The FXR agonist 6-ethyl-chenodeoxycholic acid (6-ECDCA) was synthesized by Dr. Angela Zampella as described [31].

### Animals

Protocols were approved by the University of Perugia's Animal Care Committee according to the Italian guideline for care and use of laboratory animals. The latest ID for this project is #98/2010-B. The authorization was released to Prof. Stefano Fiorucci, as a principal investigator, on May 19, 2010. C57BL/6 male mice were from Harlan Laboratories, while the colony of C57BL/6j FXR null mice was established from animals originally donated by Dr. Gonzalez F.J. (Laboratory of Metabolism, National Cancer Institute, National Institutes of Health, Bethesda, MD 20892, USA - fjgonz@helix.nih.gov) [32]. Colitis was induced in mice by intrarectal administration of TNBS (1.5 mg/mouse). The TLR-9 agonist (CpG, 10 µg/mouse) was administered i.p. in both FXR^+/+^ and FXR^−/−^ mice for 3 days before TNBS administration. The FXR agonist (6-ECDCA, 5 mg/kg) was administered i.p. to C57BL6 wild type, MyD88^−/−^ and TLR9^−/−^ mice for 3 days before TNBS administration. Animals were sacrificed 4 days after TNBS. Myeloperoxidase (MPO) activity was measured as previously described [33].

### Disease activity Index (DAI)

DAI was calculated by combining individual scores of weight loss, stool consistency and bleeding. The sum of individual scores was divided by 3 [27], [28].

### Mucosal damage score

Mucosal damage scoring was analyzed considering the presence of indurations, edema, thickness and evidence of mucosal hemorrhage, as indicated previously [19]. For histologic examination, a section of the distal colon from each animal was fixed in 10% formalin, embedded in paraffin, sectioned, and stained with Haematoxylin and Eosin (H&E).

### Cell culture

The Raw264.7, a mouse macrophage cell line, was purchased from the ATCC-LGC Standards and cultured in D-MEM supplemented with 10% Fetal Bovine serum, 1% L-glutamine and 1% penicillin/streptomycin. Serum starved Raw264.7 cells were stimulated for 18 hours with CpG ODN 2395 (2 µg/ml) in D-MEM. After stimulation cells were divided in two aliquots: 5×10^6^ cells were lysed in 1 ml TRIZOL reagent (to assess the relative mRNA expression of FXR and IRF-7 by quantitative Real-Time PCR) while 5×10^6^ cells were lysed in 500 µl SDS lysis buffer containing β-mercaptoethanol (to measure protein expression of FXR and IRF-7 by Western blotting).

### Western Blotting

Total proteins from Raw264.7 cells were separated by SDS PAGE, transferred to nitrocellulose membranes (Bio-Rad) and probed with primary antibodies FXR (Abcam), IRF-7 (SantaCruz) and α-tubulin (Sigma). The anti-immunoglobulin G horseradish peroxidase conjugate (Bio-Rad) was used as the secondary antibody and specific protein bands were visualized using the Super Signal West Dura reagent (Pierce), following the manufacturer's suggested protocol. Films were scanned and the densitometry analysis performed using the Image J software (NIH, Bethesda, MD, USA).

### Isolation and culture of human and mouse monocytes

Human CD14 derived Peripheral blood mononuclear cells (PBMCs) were obtained from normal individual donors from the Blood Bank Service of the University of Perugia Hospital. PBMCs were isolated by density gradient centrifugation through a Ficoll-Hypaque gradien (Pharmacia Biotech). Monocytes were isolated by positive selection using magnetic cell sorting according to the manufacturer's instructions (Miltenyi Biotec). After isolation monocytes were cultured in R-PMI and stimulated 18 hours with the following TLR ligands: (i) TLR1/2: 100 ng/ml Pam3Cys-Ser-(Lys)4.3HCl; (ii) TLR3: 100 µg/ml Polyinosinic-polycytidylic acid potassium salt (Poly IC); (iii) TLR4: 1 µg/ml Lipopolysaccharide from E. coli, Serotype R515; (iv) TLR5: 100 ng/ml Flagellin (FLIC); (v) TLR6: 100 ng/ml Macrophage stimulatory Lipopeptide 2 (MALP-2); (vi) TLR7–8: 10 µg/ml Polyuridylic acid potassium salt and (vii) TLR9: 2 µg/ml CpG ODN 2395. Mouse monocytes were obtained from the spleens of TLR9 wild-type and null mice (C57BL/6BJ6 background) following a previous described protocol [19]. After isolation primary murine monocytes were cultured in RPM-I and stimulated 18 hours with 2 µg/ml CpG ODN 2395.

### RNA extraction and nuclear receptor PCR array

Total RNA from serum starved Raw264.7 cells left untreated or stimulated with CpG ODN 2395 (2 µg/ml) was extracted with Trizol reagent (Invitrogen) and reverse transcribed with Superscript-II reverse transcriptase (Invitrogen) following the manual instructions. 25 ng cDNA was pipetted in each well of a 96 well PCR array plate (Human Nuclear Receptors and Coregulators RT2 Profiler TM PCR Array - http://www.sabiosciences.com/rt_pcr_product/HTML/PAHS-056A.html - Superarray Bioscience, Frederick,MD, USA) and amplified following the manual instructions. Genes selected for PCR analysis encode several classes of nuclear receptors. PCR analysis was carried out with the on-line software RT2 Profiler PCR Array Data Analysis (http://pcrdataanalysis.sabiosciences.com/pcr/arrayanalysis.php).

### Real-Time PCR

Methods for Real-Time PCR conditions and analysis have been described previously [34]. Primers were synthesized by MWG BIOTECH. Human (h) and murine (m) sense and antisense primers were as following: hFXR: tacatgcgaagaaagtgtcaaga and actgtcttcattcacggtctgat; hTNFα: aacctcctctctgccatcaa and ggaagacccctcccagatag; hGAPDH: gaaggtgaaggtcggagt and catgggtggaatcatattggaa; mFXR:; mTNFα: acggcatggatctcaaagac and gtgggtgaggagcacgtagt; mIRF7: agccctctgctttctagtgatg and ctgcatagggttcctcgtaaac; mGAPDH: ctgagtatgtcgtggagtctac and gttggtggtgcaggatgcattg.

### FXR promoter analysis, plasmid construction and Luciferase assay

Human and murine proximal promoter regions of FXR were analyzed with the on-line software TFsearch (http://www.cbrc.jp/research/db/TFSEARCH.html) for the search of putative IRF7 consensus sequences (GAA (A/T) N (C/T) GAAAN (T/C)). For luciferase assay, three tandem repeats of the putative IRF7 responsive sequence (IRF7RE) were cloned KpnI-XhoI into the pGL4 luciferase reporter vector (pGL3(IRF7RE)_3X_) using the following oligonucleotides: ACTGGGTACCCCT*GAATATCAAAGC*TGCCCT*GAATATCAAAGC*TGCCCT*GAATATCAAAGC*TGCCTCGAGACTG and CAGTCTCGAGGCAGCTTTGATATTCAGGGCAGCTTTGATATTCAGGGCAGCTTTGATATTCAGGGGTACCCAGT. 24 h before transfection, 10×10^5^ Raw264.7 cells were plated in six-well plates and cultured in D-MEM. Subsequently, cells were transiently transfected using 1 µg pGL4(IRF7RE)_3X_ and 200 ng pCMV-βgalactosidase as an internal control for transfection efficiency. Transfections were performed with the reagent FuGENE HD (Promega). Forty-eight hours post-transfection, cells were stimulated 18 hours with a dose response of CpG ODN 2395 (0.5, 1, 2 and 4 µg/ml). Control cultures received vehicle (0.1% DMSO) alone. Cells were lysed in 100 µL diluted reporter lysis buffer (Promega), and 10 µL of cellular lysate was assayed for luciferase activity using the Glomax 20/20 luminometer (Promega, Milan, Italy). Luciferase activities were normalized for transfection efficiencies by dividing the relative light units (RLU) by β-galactosidase activity.

### Electrophoretic Mobility Shift Assay (EMSA)

Nuclear extracts from serum starved Raw264.7 cells left untreated or stimulated 18 hours with CpG ODN 2395 (2 µg/ml) were prepared using the NE-PER kit (Pierce). Nuclear extracts (10 µg) were incubated for 20 min at room temperature with 20 femtomoles of biotin labeled IRF7RE wild type probe (GCCTGAATATCAAAGCTGCA) or with IRF7-RE mutated probe (GCCTGAACATCACCGCTGCA, mutated bases are shown in bold), prior to electrophoresis. For competition experiments, 100 fold excess of unlabeled probes or anti-IRF7 antibody (Santa Cruz) were incubated for 20 min with nuclear extracts from stimulated cells before addition of the biotinylated probes.

### Chromatin Immunoprecipitation (ChIP)

10×10^6^ serum starved Raw264.7 cells cultured in D-MEM were stimulated 18 hours with 2 µg/ml CpG ODN 2395 or received the vehicle alone (1% DMSO). Chromatin was immunoprecipitated with an anti-IRF7 antibody (Santa Cruz, CA, USA) or with an anti-IgG as negative control. Detailed methods for ChIP protocol and Real-Time data analysis have been previously described [35]. The sequences of primers used for the amplification of the murine FXR promoter were: gcctatgtacgtgttcattgtcc and aggaggagccaatgtttctga.

### Statistic analysis

All values are ± Standard Error (SE) of *number (n)* observations per group. Comparisons of more than two groups were made with a one-way ANOVA with post-hoc Tukey's test. Comparison of two groups was made by the Student's t-test for unpaired data when appropriate.

## Supporting Information

Table S1 **Analysis of FXR gene expression and severity of TNBS colitis in TLR2^−/−^, TLR4^−/−^, TLR9^−/−^, MyD88^−/−^ and FXR^−/−^ mice in comparison with C57/BL6 mice administered TNBS.**
(DOC)

Figure S1 **Schematic representation of TLR9/MyD88/IRF7 pathway leading to FXR gene activation.**
(TIF)

## References

[1] Beutler B (2009) Microbe sensing, positive feedback loops, and the pathogenesis of inflammatory diseases. Immunol Rev 227: 248–263. 19120489 10.1111/j.1600-065X.2008.00733.x PMC2713013

[2] Kawai T, Akira S (2010) The role of pattern-recognition receptors in innate immunity: update on Toll-like receptors. Nat Immunol 11: 373–384. 20404851 10.1038/ni.1863

[3] Cario E (2010) Toll-like receptors in inflammatory bowel diseases: a decade later. Inflamm Bowel Dis 16: 1583–1597. 20803699 10.1002/ibd.21282 PMC2958454

[4] Chiang JY (2002) Bile acid regulation of gene expression: roles of nuclear hormone receptors. Endocr Rev 23: 443–463. 12202460 10.1210/er.2000-0035

[5] Szanto A, Roszer T (2008) Nuclear receptors in macrophages: a link between metabolism and inflammation. FEBS Lett 582: 106–116. 18022390 10.1016/j.febslet.2007.11.020

[6] Kau AL, Ahern PP, Griffin NW, Goodman AL, Gordon JI (2011) Human nutrition, the gut microbiome and the immune system. Nature 474: 327–336. 21677749 10.1038/nature10213 PMC3298082

[7] Mazmanian SK, Liu CH, Tzianabos AO, Kasper DL (2005) An immunomodulatory molecule of symbiotic bacteria directs maturation of the host immune system. Cell 122: 107–118. 16009137 10.1016/j.cell.2005.05.007

[8] Guarner F (2006) Prebiotics and mucosal barrier function. J Nutr 136: 2269 author reply 2270–2271. 16857852 10.1093/jn/136.8.2269

[9] Wu GD (2007) Nuclear hormone receptors and intestinal inflammation. Gastroenterology 133: 1068. 17919480 10.1053/j.gastro.2007.08.052

[10] Bassaganya-Riera J, Reynolds K, Martino-Catt S, Cui Y, Hennighausen L, et al (2004) Activation of PPAR gamma and delta by conjugated linoleic acid mediates protection from experimental inflammatory bowel disease. Gastroenterology 127: 777–91. 15362034 10.1053/j.gastro.2004.06.049

[11] Kelly D, Campbell JI, King TP, Grant G, Jansson EA, et al (2004) Commensal anaerobic gut bacteria attenuate inflammation by regulating nuclear-cytoplasmic shuttling of PPAR-gamma and RelA. Nat Immunol 5: 104–112. 14691478 10.1038/ni1018

[12] Ricote M, Li AC, Willson TM, Kelly CJ, Glass CK (1998) The peroxisome proliferator-activated receptor-gamma is a negative regulator of macrophage activation. Nature 391: 79–82. 9422508 10.1038/34178

[13] Joseph SB, Castrillo A, Laffitte BA, Mangelsdorf DJ, Tontonoz P (2003) Reciprocal regulation of inflammation and lipid metabolism by liver X receptors. Nat Med 9: 213–219. 12524534 10.1038/nm820

[14] Fiorucci S, Rizzo G, Donini A, Distrutti E, Santucci L (2007) Targeting farnesoid X receptor for liver and metabolic disorders. Trends Mol Med 13: 298–309. 17588816 10.1016/j.molmed.2007.06.001

[15] Wildenberg ME, van den Brink GR (2011) FXR activation inhibits inflammation and preserves the intestinal barrier in IBD. Gut 60: 432–433. 21270116 10.1136/gut.2010.233304

[16] Gadaleta RM, van Erpecum KJ, Oldenburg B, Willemsen EC, Renooij W, et al (2011) Farnesoid X receptor activation inhibits inflammation and preserves the intestinal barrier in inflammatory bowel disease. Gut 60: 463–472. 21242261 10.1136/gut.2010.212159

[17] Nijmeijer RM, Gadaleta RM, van Mil SW, van Bodegraven AA, Crusius JB, et al (2011) Farnesoid X receptor (FXR) activation and FXR genetic variation in inflammatory bowel disease. PLoS One 6: e23745. 21887309 10.1371/journal.pone.0023745 PMC3161760

[18] Kim MS, Shigenaga J, Moser A, Feingold K, Grunfeld C (2003) Repression of farnesoid X receptor during the acute phase response. J Biol Chem 278: C8988–C8995. 10.1074/jbc.M212633200 12519762

[19] Vavassori P, Mencarelli A, Renga B, Distrutti E, Fiorucci S (2009) The bile acid receptor FXR is a modulator of intestinal innate immunity. J Immunol 183: 6251–6261. 19864602 10.4049/jimmunol.0803978

[20] Rachmilewitz D, Karmeli F, Takabayashi K, Hayashi T, Leider-Trejo L, et al (2002) Immunostimulatory DNA ameliorates experimental and spontaneous murine colitis. Gastroenterology 122: 1428–1441. 11984528 10.1053/gast.2002.32994

[21] Rachmilewitz D, Katakura K, Karmeli F, Hayashi T, Reinus C, et al (2004) Toll-like receptor 9 signaling mediates the anti-inflammatory effects of probiotics in murine experimental colitis. Gastroenterology 126: 520–528. 14762789 10.1053/j.gastro.2003.11.019

[22] Honda K, Yanai H, Negishi H, Asagiri M, Sato M, et al (2005) IRF-7 is the master regulator of type-I interferon-dependent immune responses. Nature 434: 772–777. 15800576 10.1038/nature03464

[23] Fiorucci S, Cipriani S, Mencarelli A, Renga B, Distrutti E, et al (2010) Counter-regulatory role of bile acid activated receptors in immunity and inflammation. Curr Mol Med 10: 579–595. 20642438 10.2174/1566524011009060579

[24] Glass CK, Saijo K (2010) Nuclear receptor transrepression pathways that regulate inflammation in macrophages and T cells. Nat Rev Immunol 10: 365–376. 20414208 10.1038/nri2748

[25] Ogawa S, Lozach J, Benner C, Pascual G, Tangirala RK, et al (2005) Molecular determinants of crosstalk between nuclear receptors and toll-like receptors. Cell 122: 707–721. 16143103 10.1016/j.cell.2005.06.029 PMC1430687

[26] Fiorucci S, Rizzo G, Antonelli E, Renga B, Mencarelli A, et al (2005) A farnesoid x receptor-small heterodimer partner regulatory cascade modulates tissue metalloproteinase inhibitor-1 and matrix metalloprotease expression in hepatic stellate cells and promotes resolution of liver fibrosis. J Pharmacol Exp Ther 314: 584–595. 15860571 10.1124/jpet.105.084905

[27] Fiorucci S, Mencarelli A, Palazzetti B, Sprague AG, Distrutti E, et al (2002) Importance of innate immunity and collagen binding integrin alpha1beta1 in TNBS-induced colitis. Immunity 17: 769–780. 12479823 10.1016/s1074-7613(02)00476-4

[28] Sato M, Suemori H, Hata N, et al (2000) Distinct and essential roles of transcription factors IRF-3 and IRF-7 in response to viruses for IFN-alpha/beta gene induction. Immunity 13: 539–548. 11070172 10.1016/s1074-7613(00)00053-4

[29] Zhang L, Pagano JS (2002) Structure and function of IRF-7. J Interferon Cytokine Res 22: 95–101. 11846980 10.1089/107999002753452700

[30] Mencarelli A, Distrutti E, Renga B, D'Amore C, Cipriani S, et al (2011) Probiotics modulate intestinal expression of nuclear receptor and provide counter-regulatory signals to inflammation-driven adipose tissue activation. PLoS One 6: e22978. 21829567 10.1371/journal.pone.0022978 PMC3146529

[31] Sepe V, Ummarino R, D'Auria MV (2012) Conicasterol E, a Small Heterodimer Partner Sparing Farnesoid X Receptor Modulator Endowed with a Pregnane X Receptor Agonistic Activity, from the Marine Sponge Theonella swinhoei. J Med Chem 55: 84–93. 22126372 10.1021/jm201004p

[32] Sinal CJ, Tohkin M, Miyata M, Ward JM, Lambert G, et al (2000) Targeted disruption of the nuclear receptor FXR/BAR impairs bile acid and lipid homeostasis. Cell 102: 731–744. 11030617 10.1016/s0092-8674(00)00062-3

[33] Santucci L, Fiorucci S, Di Matteo FM, Morelli A (1995) Role of tumor necrosis factor alpha release and leukocyte margination in indomethacin-induced gastric injury in rats. Gastroenterology 108: 393–401. 7530670 10.1016/0016-5085(95)90065-9

[34] Renga B, Migliorati M, Mencarelli A, Cipriani S, D'Amore C, et al (2011) Farnesoid X receptor suppresses constitutive androstane receptor activity at the multidrug resistance protein-4 promoter. Biochim Biophys Acta 1809: 157–165. 21296199 10.1016/j.bbagrm.2011.01.008

[35] Renga B, Mencarelli A, D'Amore C, Cipriani S, Baldelli F, et al (2012) Glucocorticoid receptor mediates the gluconeogenic activity of the farnesoid X receptor in the fasting condition. FASEB J 26: 3021–31. 22447981 10.1096/fj.11-195701

