# Supplementary material for: The Bile Acid Sensor FXR Is Required for Immune-Regulatory Activities of TLR-9 in Intestinal Inflammation
Source: PLoS One. 2013 Jan 25;8(1):e54472. doi: 10.1371/journal.pone.0054472 (PMC3555871; doi:10.1371/journal.pone.0054472)
Supplement: Table S1 — Analysis of FXR gene expression and severity of TNBS colitis in TLR2−/−, TLR4−/−, TLR9−/−, MyD88−/− and FXR−/− mice in comparison with C57/BL6 mice administered TNBS. (DOC) [file pone.0054472.s001.doc]

Table S1. Analysis of FXR gene expression and severity of TNBS colitis in TLR2^-/-^, TLR4^-/-^, TLR9^-/-^, MyD88^-/-^ and FXR^-/-^ mice in comparison with C57/BL6 mice administered TNBS.

| **KO phenotype vs C57/BL6** | **Severity of colitis** | **FXR gene expression** |
| --- | --- | --- |
| **TLR2^-/-^** | ↔ | ↑ |
| **TLR4^-/-^** | ↓↓ | ↔ |
| **TLR9^-/-^** | ↑↑ | ↓↓ |
| **MyD88^-/-^** | ↔ | ↔ |
| **FXR^-/-^** | ↑↑↑ | ND |

Legend for severity of colitis: ↔ unchanged; ↓↓ improved; ↑↑ worsened; ↑↑↑ drastically worsened. Legend for FXR gene expression : ↑ slightly induced; ↔ unchanged; ↓↓ strongly downregulated; ND, not detectable.
