# Supplementary figures and images for: The Bile Acid Sensor FXR Is Required for Immune-Regulatory Activities of TLR-9 in Intestinal Inflammation
Source: PLoS One. 2013 Jan 25;8(1):e54472. doi: 10.1371/journal.pone.0054472 (PMC3555871; doi:10.1371/journal.pone.0054472)

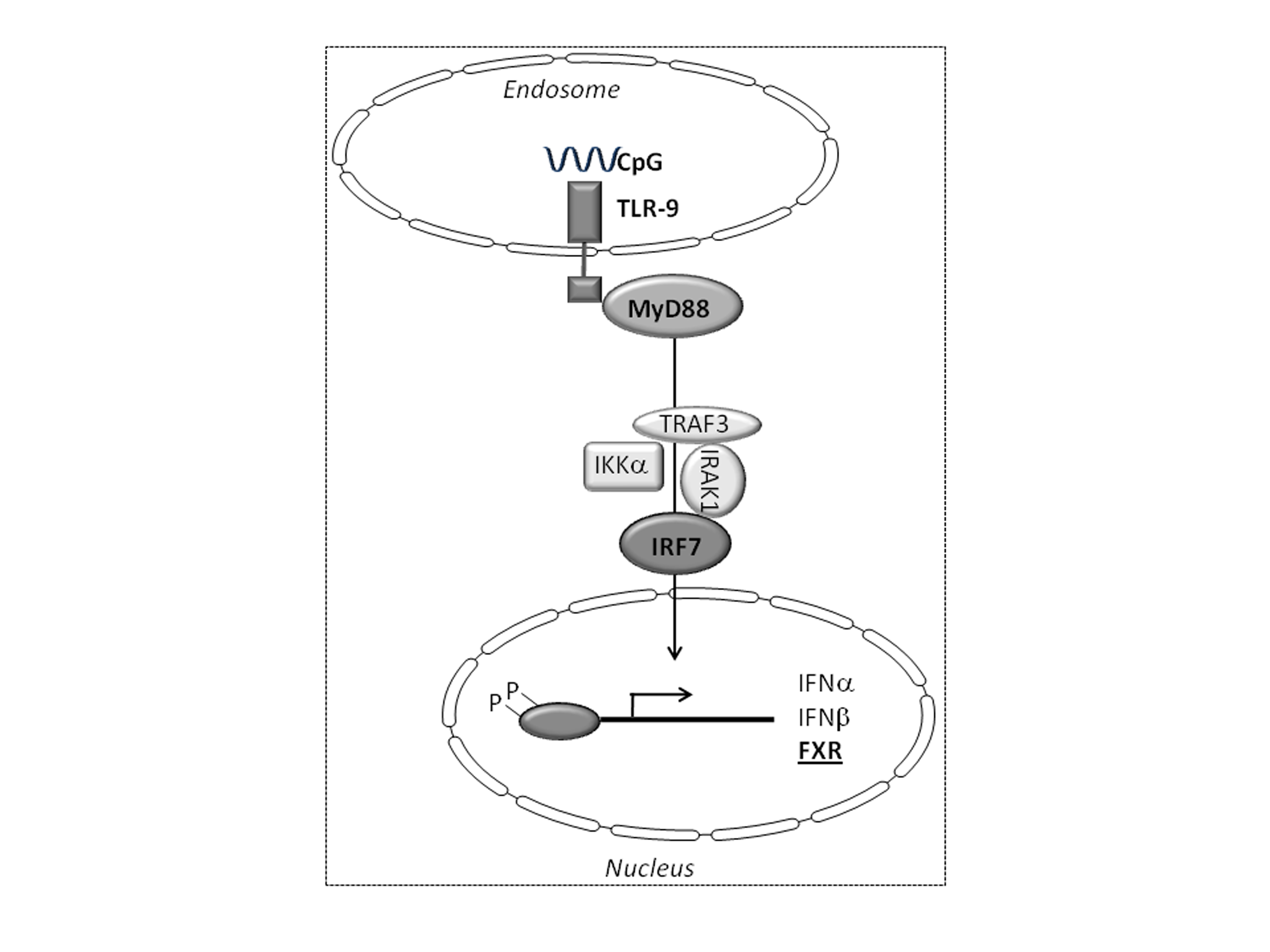

Supplement: Figure S1 — Schematic representation of TLR9/MyD88/IRF7 pathway leading to FXR gene activation. (TIF) [file pone.0054472.s002.tif]
